# Limited performance questions retrospective use of quantitative flow ratio in coronary artery bypass grafting

**DOI:** 10.3389/fcvm.2026.1757011

**Published:** 2026-02-02

**Authors:** Hannes Abfalterer, Dominik Janker, Lorenz Rüf, Yannik Reiter, Sarah Maier, Nikolaos Bonaros, Michael Grimm, Axel Bauer, Elfriede Ruttmann-Ulmer

**Affiliations:** 1 Department of Cardiac Surgery, Medical University of Innsbruck, Innsbruck, Austria; 2 Institute of Clinical Epidemiology, Public Health, Health Economics, Medical Statistics and Informatics, Medical University of Innsbruck, Innsbruck, Austria; 3 Department of Cardiology, Medical University of Innsbruck, Innsbruck, Austria

**Keywords:** arterial grafts, CABG, patency rate, QFR, retrospective

## Abstract

**Background:**

Hemodynamic assessment of coronary artery stenosis has impact on arterial graft patency. Quantitative flow ratio (QFR) obtains hemodynamic information of coronary artery stenosis.

**Methods:**

Patients with history of isolated coronary artery bypass grafting (with ≥1 arterial graft) and at least one postoperative coronary re-assessment were retrospectively investigated. The preoperative angiography was used for retrospective QFR analysis of the native coronary target vessel, to which the arterial bypass graft was anastomosed. Analysis was performed by certified investigators, who were blinded towards postoperative arterial graft patency status. Coronary targets with QFR values of ≤0.80 were defined as hemodynamically relevant, whereas values of >0.80 were defined as hemodynamically irrelevant.

**Results:**

Out of 5,692 patients, 596 patients had a postoperative coronary assessment and were therefore eligible for inclusion. In 196 arterial target vessels QFR analysis was possible. Kaplan–Meier analysis revealed higher graft patency rates for arterial grafts anastomosed to coronary branches with QFR values ≤0.80 (log-rank: *p* = 0.017). In multivariable Cox regression analysis, QFR ≤ 0.80 remained an independent predictor for arterial graft patency (HR: 0.475, 95% CI: 0.261–0.867; *p* = 0.015), while visually estimated stenosis from preoperative coronary angiography did not (*p* = 0.160). With an area under the curve of 0.595, 95% CI (0.503–0.688), the performance of the model was poor to at most moderate. Most target vessels [546 (80.22%)] were not analysable in retrospective fashion.

**Conclusion:**

Though target vessel QFR ≤ 0.80 was associated with higher arterial graft patency, our trial observed low feasibility (high drop out rates) and poor diagnostic performance of QFR used in retrospective fashion. Caution is warranted for retrospective use of QFR in datasets with similar constraints.

## Introduction

Various studies have demonstrated the importance of functional assessment of coronary artery disease severity in patients undergoing coronary stent implantation (1–3). Current guidelines recommend the use of fractional flow reserve (FFR), instantaneous wave free ratio (iFR) and quantitative flow ratio (QFR) to assess functional severity of intermediate diameter stenosis and as a guidance for revascularization (4).

On the other hand, data on functional assessment of coronary artery stenosis severity in coronary artery bypass grafting (CABG) is both limited and contradictory (5). While several trials (6–8) demonstrated an impact of preoperative functional testing using FFR on arterial graft patency rates, the Fargo trial (9) and GRAFFITI trial (10) were negative. None of the trials has shown an association of preoperative functional testing on graft patency rates in venous bypass grafts. Thus, current evidence suggests that functional testing using FFR may help in defining indications for the use of arterial grafts in CABG (6).

FFR itself is an invasive assessment, requiring intracoronary pressure wire application.

QFR is a technique, which uses two angiographic projections for a 3-dimensional reconstruction and functional assessment of coronary artery disease severity (11). This method has shown sufficient correlation with respect to FFR and iFR measurements. Furthermore, QFR measurement is not dependent on intracoronary pressure wire application or adenosine, therefore being safer and more time effective.

Wang et al. retrospectively analysed the effect of QFR values on graft patency rates of internal thoracic arteries grafted to the left anterior descending artery (LAD) and concluded, that a QFR value > 0.80 was a risk factor for graft occlusion (12). Hu et al. showed that QFR outperformed the commonly used angiographic degree of stenosis in predicting radial artery (RA) graft occlusion, with a best cut-off value of 0.71 (13). Wang et al. further demonstrated, that QFR serves as a valuable tool for selecting the appropriate conduit for specific coronary vessels (in this case, RA grafts achieved superior patency, compared to saphenous vein grafts, in vessels with QFR values ≤0.57) (14).

## Materials and methods

Permission for this investigation was approved by the local institutional review board. Patients informed consent was waived. (Medical University Innsbruck, January 24th 2024, Number: 1360/2023).

Patients’ who underwent isolated CABG at the Department of Cardiac Surgery at the Medical University of Innsbruck (between 2004 and 2022) and received at least one arterial graft, were followed in a retrospective database. Of them, we isolated patients who underwent angiographic follow-up [either with invasive coronary angiography or coronary CT angiography (cCTA)]. In case of multiple re-imaging examinations, the first one occurring after surgery was used for statistical analysis. Sequential grafts were excluded from analysis. QFR analysis was performed with Medis QFR CE® by Medis Medical Imaging (2316 XG Leiden, The Netherlands). Three certified investigators, blinded for postoperative arterial graft patency status, performed the QFR analysis. In case the QFR analysis was not possible, reasons were documented. In case of feasible QFR measurements, the analysis was performed on the coronary artery branch, to which the arterial graft was anastomosed. Contrast flow QFR values were documented. Coronary artery stenoses with QFR values ≤0.80 were defined as hemodynamically relevant, whereas those with QFR values > 0.80 were defined as hemodynamically irrelevant. Graft dysfunction was defined as ≥50% stenosis or string sign. Graft occlusion was defined as absence of contrast medium in the lumen of the graft. All other grafts were defined as patent.

Baseline demographic data (patient level- and graft level analysis) are presented as mean ± standard deviation (SD) or as median + interquartile range for continuous variables and as number (%) for categorical variables. Testing for statistical differences between the QFR cohorts (QFR ≤ 0.80 and QFR > 0.80) was done by applying two-sample *t*-test, Mann–Whitney *U*-Test, chi-square test or Fisher`s exact test, where applicable. Kaplan–Meier analysis with log-rank testing was performed to compare graft patency rates between the two QFR cohorts (QFR ≤ 0.80 and QFR > 0.80). A shared frailty model was used in Cox-regression analysis to identify independent predictors for graft dysfunction/occlusion (using hazard ratios, 95% CI and *p*-values) and to adjust for clustering of grafts within the same patients. Only variables which showed statistical significance (*p* < 0.05) in univariate analysis were included in this multivariable model. Receiver operating characteristics (ROC) curve analysis was used to identify cut-off values with corresponding sensitivity/specificity and area under the curve (AUC).

*P*-values < 0.05 were defined to be statistically significant.

All statistical analyses were performed using IBM SPSS Statistics Version 30.0.0.0 (IBM Corporation, 1 New Orchard Road, Armonk, NY 10504-1722, United States), R Version 4.5.1 (R Foundation for Statistical Computing, Vienna, Austria), RStudio Version 2025.0.1 + 513 (Posit, Boston, Massachusetts, United States of America) and MedCalc Statistical Software Version 19.0.5 (MedCalc Software Ltd., Ostend, Belgium).

## Results

Out of 5,692 patients receiving isolated CABG between 2004 and 2022, we identified 596 patients with 774 arterial grafts [351 left internal thoracic arteries (LITA)/ 184 right internal thoracic arteries (RITA)/ 239 RA] who were eligible for inclusion. 31 sequential grafts (20 LITA and 11 RA) were excluded, resulting in 743 remaining arterial grafts.

546 (80.22%) of the target vessels could not be analysed for the following reasons:

- Target vessel occlusion (with or without collateralization): 47
- Severe vessel overlap with other coronary vessels: 196
- Poor angiographic image quality: 52
- Lack of a second projection in >25° angle: 12
- Ostial stenosis of target vessel: 24
- Extensive foreshortening: 6
- Technical issues (due to missing calibration data): 209

One graft with perioperative graft dysfunction for surgical reasons was excluded from the analysis.

177 patients with 196 arterial grafts (82 LITA/48 RITA/66 RA) and their coronary targets were analysable.

Supplementary Figure 1 represents a flow-chart to illustrate the selection process of eligible cases for analysis.

Table 1 depicts baseline characteristics between the two QFR cohorts (QFR ≤ 0.80 and QFR > 0.80) on patient-level basis. Baseline characteristics were balanced between the two QFR cohorts, but patients with QFR > 0.80 more often had previous percutaneous coronary intervention (PCI) (*p* = 0.017), however previous PCI of the target vessel did not show any difference (*p* = 0.886).

**Table 1:** Baseline characteristics.

Variable (patient level analysis)	QFR ≤ 0.80 (*n* = 109)	QFR > 0.80 (*n* = 68)	*p*-value
Age, mean ± SD	61.59 ± 9.15	62.82 ± 8.71	0.630^1^
Female, *n* (%)	12 (11.00)	11 (16.18)	0.320^2^
Arterial hypertension, *n* (%)	91 (83.50)	53 (77.94)	0.357^2^
Hypercholesterolemia, *n* (%)	90 (82.57)	56 (82.35)	0.971^2^
Absence of diabetes mellitus, *n* (%)	77 (70.64)	55 (80.88)	0.305^2^
Insulin dependent diabetes mellitus, *n* (%)	11 (10.10)	5 (7.35)	
Not insulin dependent diabetes mellitus, *n* (%)	21 (19.26)	8 (11.76)	
Never smoker, *n* (%)	59 (54.13)	30 (44.12)	0.199^2^
Former smoker, *n* (%)	16 (14.68)	17 (25.00)	
Active smoker, *n* (%)	34 (31.20)	21 (30.89)	
Adipositas, *n* (%)	31 (28.44)	15 (22.06)	0.346^2^
Pavd, *n* (%)	9 (8.26)	10 (14.71)	0.178^2^
COPD, *n* (%)	6 (5.50)	4 (5.89)	>0.99^3^
Dialysis, *n* (%)	2 (1.83)	0 (0.00)	0.524^3^
Previous TIA, *n* (%)	1 (0.92)	0 (0.00)	>0.99^3^
Previous stroke, *n* (%)	6 (5.50)	1 (1.47)	0.252^3^
Previous ACS, *n* (%)	40 (36.70)	18 (26.47)	0.159^2^
Previous PCI, *n* (%)	21 (19.27)	24 (35.29)	**0** **.** **017** ^2^
Previous PCI of target vessel, *n* (%)	9 (8.26)	6 (8.82)	0.886^2^
Left main stem stenosis ≥50%, *n* (%)	28 (25.69)	10 (14.71)	0.227^2^

ACS, acute coronary syndrome; COPD, chronic obstructive pulmonary disease; n, number; Pavd, peripheral arterial vascular disease; PCI, percutaneous coronary intervention; SD, standard deviation; TIA, transitory ischaemic attack.

The bold values mean that this is statistically significant with *p* values <0.05.

1 Two-sample *t*-test.

2 Chi-square test.

3 Fisher's exact test.

Supplementary Table S1 depicts operative characteristics between the two QFR cohorts (QFR ≤ 0.80 and QFR > 0.80) on patient-level basis, with no significant difference in the investigated variables.

Supplementary Table S2 highlights graft specific operative data according to subgroups (LITA/RITA/RA), while Supplementary Table S3 demonstrates the distribution of grafts to coronary target vessels according to the QFR cohorts, with no significant difference in the investigated variables.

Re-imaging was performed 4.39 ± 3.88 years (QFR ≤ 0.80) vs. 4.45 ± 4.23 years (QFR > 0.80) (*p* = 0.284) after surgery.

Re-imaging was performed due to the following reasons:

- suspected progression of coronary artery disease in 126/177 (71.19%)
- acute coronary syndrome in 15/177 (8.47%)
- routine medical examination prior to a scheduled operation in 7/177 (3.95%)
- in 15/177 (8.47%) asymptomatic patients (for example due to scheduled re-imaging according to the participation in a clinical trial).

Re-imaging was performed with invasive coronary angiography in 167 (94.35%) cases and with cCTA in 10 (5.65%) cases.

Overall, 141/196 (71.94%) grafts were patent, and 55/196 (28.06%) grafts were either dysfunctional or occluded.

120/196 (61.22%) of grafts were anastomosed to coronary branches which had QFR values ≤0.80, 76/196 (38.78%) of grafts were anastomosed to coronary branches which had QFR values >0.80.

**Figure 1:**
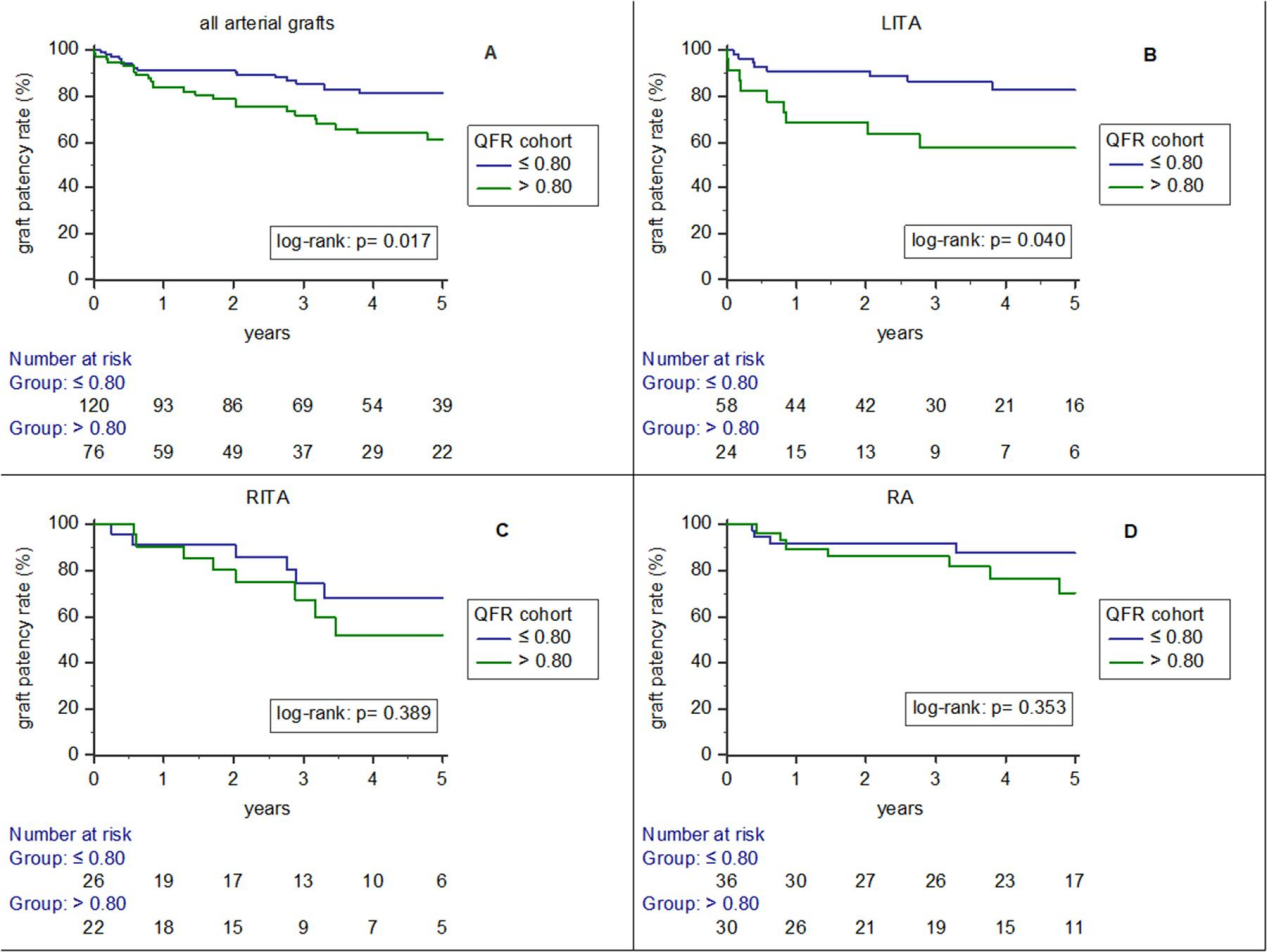
Results of Kaplan-Meier analysis on arterial graft patency rates according to QFR cohort (QFR ≤ 0.80 and QFR > 0.80) and according to analysed grafts [all arterial grafts **(A)**, LITA **(B)**, RITA **(C)** and RA **(D)**].

Figure 1 depicts results of Kaplan-Meier analysis on arterial graft patency rates according to QFR cohort (QFR ≤ 0.80 and QFR > 0.80) and according to analysed grafts [all arterial grafts (1A), LITA (1B), RITA (1C) and RA (1D)]. Graft patency rate was significantly higher for all arterial grafts (log-rank: *p* = 0.017) and LITA grafts (log-rank: *p* = 0.040)), if anastomosed to target vessels with QFR ≤ 0.80. For RITA grafts (log-rank: *p* = 0.389) and RA grafts (log-rank: *p* = 0.353) this trend was not significant.

The cumulative graft patency rates (at different time points after surgery) for all arterial grafts (from Figure 1A) in Kaplan–Meier analysis) are visualized in Supplementary Table S4.

**Figure 2:**
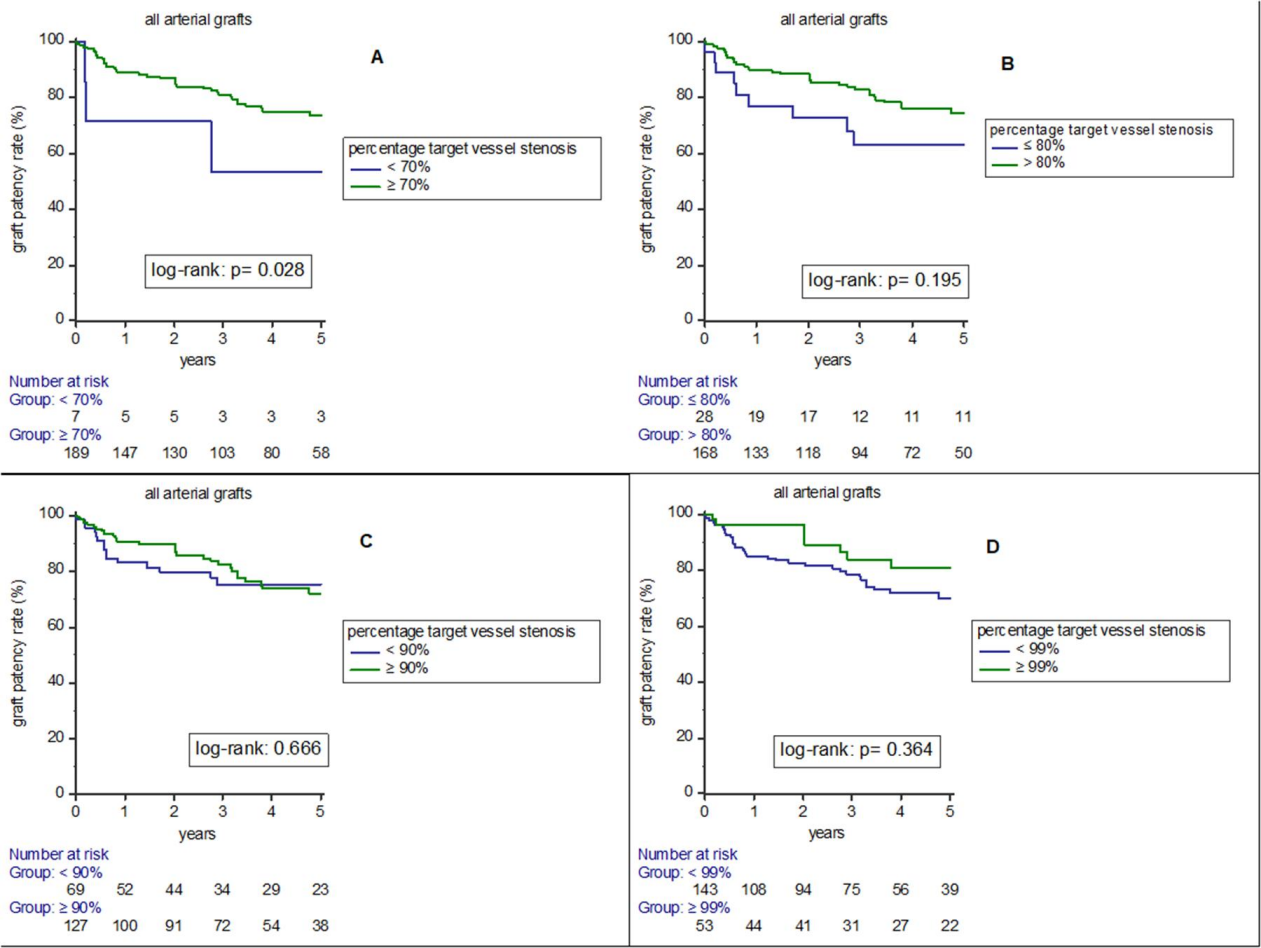
Results of Kaplan-Meier analysis on arterial graft patency rates (all arterial grafts) according to percentage target vessel stenosis subgroups [< 70% and ≥ 70% **(A)**, ≤ 80% and > 80% **(B)**, < 90% and ≥ 90% **(C)** and < 99% and ≥ 99% **(D)**].

Figure 2 represent results of Kaplan-Meier analysis on arterial graft patency rates (all arterial grafts) according to percentage target vessel stenosis subgroups. Arterial grafts bypassed to target vessels with ≥ 70% stenosis had significantly higher patency than arterial bypass grafts anastomosed to target vessels with < 70% stenosis (*p* = 0.028). For other subgroups, there was no significant difference.

**Figure 3:**
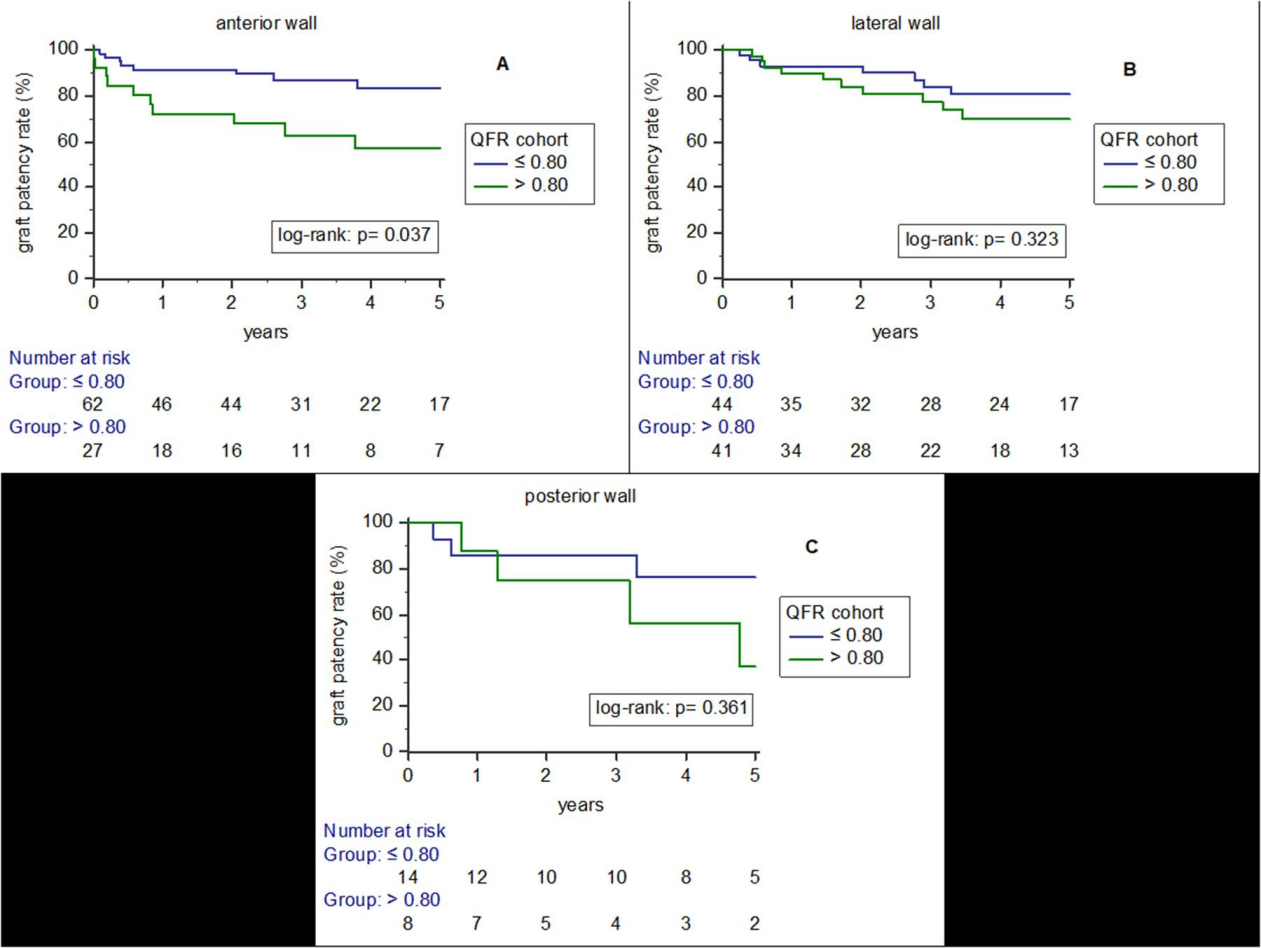
Results of Kaplan-Meier analysis on arterial graft patency rates according to QFR cohort (QFR ≤ 0.80 and QFR > 0.80) and according to target vessel territory [anterior wall **(A)**, lateral wall **(B)** and posterior wall **(C)**].

Figure 3 shows results of Kaplan-Meier analysis on arterial graft patency rates according to QFR cohort (QFR ≤ 0.80 and QFR > 0.80) and according to target vessel territory [anterior wall (3A), lateral wall (3B) and posterior wall (3C)]. Arterial grafts bypassed to hemodynamically relevant stenosed target vessels (QFR ≤ 0.80) of the anterior wall had significantly higher patency rates (*p* = 0.037). There was no significant finding for the lateral (*p* = 0.323) or posterior (*p* = 0.361) wall territory.

9 variables [QFR cohort (QFR ≤ 0.80 and QFR > 0.80), degree of stenosis ≥70% in preoperative invasive coronary angiography, on- or off-pump surgery, gender, smoking status, postoperative antiplatelet therapy, postoperative therapy with ezetimibe, postoperative therapy with proprotein convertase subtilisin/kexin type 9 inhibitor (PCSK9I) and skeletonized harvesting technique) showed significance (*p* < 0.05) in univariate analysis (see Supplementary Table S5).

Table 2 represents the results of multivariable Cox regression analysis (according to arterial graft patency). While QFR ≤ 0.80 remained a significant predictor for higher arterial graft patency rate [HR: 0.475, 95%CI (0.261–0.867), *p* = 0.015], degree of stenosis ≥ 70% in preoperative invasive coronary angiography did not remain significant (*p* = 0.160).

**Table 2:** Multivariable Cox regression analysis.

Variable	HR	95% CI	*p*-value
QFR ≤ 0.80	0.475	0.261–0.867	**0**.**015**
Visually estimated stenosis ≥70% in preop. invasive coronary angiography	0.465	0.160–1.355	0.160
Off-pump surgery	5.854	1.286–26.638	**0**.**022**
Skeletonized harvesting technique	2.591	1.342–5.003	**0**.**005**
Female	2.443	1.241–4.809	**0**.**010**
Former smoker	1.179	0.537–2.591	0.680
Active smoker	2.880	1.484–5.588	**0**.**002**
Postop. antiplatelet therapy	0.096	0.012–0.806	**0**.**031**
Postop. treatment with ezetimibe	3.305	1.477–7.397	**0**.**004**
Postop. treatment with PCSK9I	4.049	0.241–67.961	0.330
Variance of random effect	0.00005		

CI, confidence interval; HR, hazard ratio; PCSK9I, proprotein convertase subtilisin/kexin type 9 inhibitor; postop., postoperative; preop., preoperative; QFR, quantitative flow ratio.

The bold values mean that this is statistically significant with *p* values <0.05.

ROC-curve analysis (for all arterial grafts) revealed an AUC of 0.595, 95% CI (0.503–0.688) (*p* = 0.044), with a sensitivity of 54.5% and specificity of 68.1% (at a cut-off value of 0.8050).

For internal thoracic arteries (LITA + RITA) the AUC was higher [0.638, 95% CI (0.524–0.752), *p* = 0.018] than for RA grafts alone [0.501, 95% CI (0.344–0.658), *p* > 0.99].

The results of ROC-curve analysis (overall and stratified analysis) are depicted in Table 3.

**Table 3:** ROC-curve analyses.

Graft type	AUC	95% CI	*p*-value	Sensitivity (%)	Specificity (%)
All grafts	0.595	0.503–0.688	0.044	54.5	68.1
LITA + RITA	0.638	0.524–0.752	0.018	48.7	84.6
RA	0.501	0.344–0.658	>.99	56.3	58

AUC, area under the curve; CI, confidence interval; LITA, left internal thoracic artery; RA, radial artery; RITA, right internal thoracic artery.

### Comment

The following are the findings of this investigation:

1. QFR value ≤0.80 is an independent predictor for higher arterial graft patency (findings mostly driven by LITA grafts respectively the anterior wall territory).
2. visual estimation of coronary artery stenosis severity did not remain a predictor for arterial graft patency

however, more importantly:

3. the feasibility of retrospective QFR analysis in this dataset was highly limited (80.22% of coronary target vessels not analysable), mainly due to projection related issues,
4. and the overall discriminatory potential was mostly poor (even if more effective for internal thoracic arteries than RA grafts).

Our investigation highlights, that arterial graft patency rate is dependent on the hemodynamic relevance of the target vessel stenosis (in a highly selected patient cohort). Arterial grafts, which have been anastomosed to target vessels with QFR value ≤0.80 had significantly higher patency rates (in comparison to QFR > 0.80). This effect was significant not only in univariate but remained so in multivariable analysis. These findings are similar to other previous publications. Wang et al. (12) demonstrated that internal thoracic arteries anastomosed to LAD with QFR > 0.80 had significantly higher risk of occlusion. Hu et al. (13) and Wang et al. (14) further showed that RA grafts reveal higher patency rates if anastomosed to functional relevantly stenosed coronary vessels, however revealed to have much lower cut-off values. Tian et al. (15) found that target vessel QFR > 0.80 was significantly associated with arterial graft occlusion but not with vein graft occlusion at 12 months. As depicted from Kaplan–Meier curves in the 3 subgroups (LITA/RITA/RA), in univariate analysis, all 3 graft types show the same trend (higher graft patency rate for grafts anastomosed to vessels with QFR value ≤0.80), however only for LITA grafts this trend was statistically significant (*p* = 0.040). The non-significant results for RITA and RA grafts might be attributed to the smaller sample size for RITA and RA grafts and for RA grafts especially due to the fact, that these graft`s patency rate might more relevantly relate on the hemodynamic situation (potentially lower cut-off values) than internal thoracic arteries do.

Visual estimation of coronary artery stenosis severity from preoperative invasive coronary angiography did not remain a predictor for arterial graft patency status, as we were not able to show any factor (only one of five factors showed significant results in univariate analysis) to remain significant in multivariable analysis. These findings are also in concordance with results of other investigations. The publications by Hu et al. (13) and Wang et al. (14) show that QFR outperforms visual estimation in the prediction of arterial graft patency status.

Most of eligible grafts were not analysable in this investigation. Common reasons were projection related issues (260/546) and technical issues (209/546). Projection related issues are related to the fact, that coronary angiography projections were not made in the intention to perform QFR analysis in the future. Frame rates of 7.5 frames/second were frequently used, possibly resulting in worse image quality. Coronary angiographies from the early study period (mostly 2004–2007) had missing calibration data (technical issue), therefore precluding QFR analysis. Other trials depict lower rates of not-analysable vessels than our trial. A small prospective trial including 22 patients reports 9.72% of vessels to be not analysable (16). The PRIDE-METAL registry (prospective, multi-centre, observational study) observed an analysability of 85.5% (17). In the retrospective analysis of Tian et al. (15), which has also been commented on in the editorial commentary by O'Brien et al. (18), states that 23.1% of participants had to be excluded from the analysis. One trial (19) used QFR values of 0.50 as an imputation for all vessels with total or subtotal occlusion, which is another common reason (47/546) for not analysability in our investigation. By excluding technical issues (in angiograms mostly from 2004 to 2007) and excluding occluded target vessels (QFR measurement generally not possible) our investigation would still report rates of non-analysability in almost 40% of target vessels. This highlights that QFR analysis is largely not feasible for retrospective analysis in datasets with similar constraints to ours.

In ROC curve analysis (all arterial grafts), with an AUC of 0.595, 95% CI (0.503–0.688), we observed a poor to at most moderate diagnostic performance. The diagnostic performance was more effective by only analysing LITA and RITA grafts combined [AUC = 0.638 95%CI (0.524–0.752)] and less effective by only analysing RA grafts [AUC = 0.501 95%CI (0.344–0.658)]. This heterogeneous result across different arterial graft types suggests that graft-specific features (possibly different cut-off values for internal thoracic arteries vs. RA grafts) influence the accuracy of the diagnostic performance of the analysis.

An optimal cut-off value of 0.8050 (close to the PCI derived cut-off value of 0.80) is most likely unsuitable for CABG. Most of current evidence (using different modalities for hemodynamic assessment) supports lower cut-off values for arterial bypass graft patency. Glineur et al. (6) saw higher arterial graft patency if coronary target vessels had FFR values ≤0.78. Buske et al. (20) observed a cut-off value of 0.75 using angiography-derived vessel FFR. The previously mentioned investigations by Hu et al. (13) and Wang et al. (14) show even lower cut-off values for radial artery graft patency (QFR 0.71/QFR ≤ 0.57).

### Limitations

This is a retrospective, single-centre analysis in a highly selected patient population (the analysable cohort is extremely small in relation to the original CABG population, due to indication-driven re-imaging). Mainly patients with symptoms received re-angiography, resulting in selection bias and under-detection of silent graft failure. Furthermore, QFR analysis was performed retrospectively on coronary angiographies that were not intended to be used for this analysis in the future. This resulted in a high rate of angiographies that were not analysable. Additionally, difficult-to-image vessels were systematically excluded, possibly resulting in the overrepresentation of easy-to-assess images. Angiographies were performed with frame rates of 7.5 frames/second frequently, which is lower than the number of frames/second specified by the company for QFR analysis (15 frames/second), possibly resulting in worse image quality. As it seems that different types of arterial grafts might have different cut-off values (possibly lower for RA grafts than for internal thoracic arteries), choosing a defined QFR value (in our case ≤0.80 vs. > 0.80) might reduce the diagnostic power of QFR analysis. The current analysis further lacks information on intraoperative flow measurement as a tool to identify grafts that were dysfunctional/occluded due to surgical issues intraoperatively. Also, intraoperative information on anastomotic quality, graft quality, distal run-off and competitive flow is unavailable. In this investigation, we did not analyse venous bypass grafts, therefore we cannot make any suggestions on the impact of QFR analysis on graft patency rates in venous grafts nor on the impact of QFR analysis on arterial vs. venous graft patency rate. The inclusion period (2004–2022) is long, possibly resulting in changes in surgical and imaging standards over time.

### Strengths

Investigators underwent certification prior to QFR analysis. Investigators were blinded towards postoperative graft patency status potentially mitigating assignment bias.

### Future prospects

Information is cumulating on the importance of functional assessment of coronary artery stenosis severity. Functional assessment of coronary artery stenosis severity seems to be especially important for arterial graft patency and therefore might be a strong instrument for the selection of arterial target vessels.

Our results must be interpreted as dataset-specific (with the above mentioned technical and methodological limitations of our retrospective dataset, resulting in low feasibility and poor diagnostic performance). Therefore, we do not generally discourage from retrospective use of QFR, however advise against retrospective use of QFR, if datasets with similar technical constraints are used. In contrast to another retrospective analysis (12), which describes higher diagnostic performance [AUC 0.76, 95%CI (0.68–0.84)], our dataset differs in various aspects:

-Lower frame rates in preoperative angiography in our dataset (mostly around 7.5 f/s vs. 15 f/s)
-Older angiograms (beginning in 2004)
-Angiograms acquired without intend to perform QFR analysis in the future

Therefore, defining characteristics of an ideal retrospective dataset would be helpful to increase feasibility and diagnostic power if QFR was used retrospectively in future research. The following points are of particular importance:

-Angiographic images must be made in accordance to manufacturer-specific requirements (frame rates of ≥15 f/s, with appropriate projections),
-Therefore, angiographic images must be performed with the intend to use them for QFR analysis in the future,
-Exclusion of lesion types known to be unsuitable for QFR analysis (ostial lesions, chronic total occlusion, bifurcation lesions and left main stenosis)

However, it seems that such requirements would result in datasets being generated in prospective way, only being analysed retrospectively afterwards, rather than representing a purely retrospective analysis (as it was the case in our dataset).

Since QFR is solely based on invasive coronary angiography, it is therefore not able to add additional information on morphological aspects of coronary artery disease such as cCTA does. As described by Feuchtner et al. (21) this morphological aspect has important prognostic value for the prediction of future major adverse cardiac events (MACE). Furthermore, as previously published by Hansson et al. (22), a retrospective analysis of the SWEDEHEART registry (including over 18,000 patients) showed that patients with preoperative physiological stenosis assessment received fewer distal anastomoses [this has been described in the GRAFFITI trial as well (10)] and had a higher risk for new angiography and new revascularization after 2 years after CABG. Therefore, functional assessment of coronary artery stenosis severity might guide the decision for arterial vs. venous graft selection (more trials needed), but one should be cautious on deferring hemodynamically non-significant leasons, since coronary disease might further progress and lead to MACE in the future.

## Conclusion

Although QFR ≤ 0.80 was associated with higher arterial graft patency rate, our trial observed low feasibility (high drop out rates) and poor diagnostic performance of QFR in a pure retrospective fashion. Therefore, caution is warranted for retrospective use of QFR in datasets with similar constraints.

## Data Availability

The raw data supporting the conclusions of this article will be made available by the authors, without undue reservation.
