# Supplementary material for: Limited performance questions retrospective use of quantitative flow ratio in coronary artery bypass grafting
Source: Front Cardiovasc Med. 2026 Feb 2;13:1757011. doi: 10.3389/fcvm.2026.1757011 (PMC12907413; doi:10.3389/fcvm.2026.1757011)
Supplement: Supplementary file 1 [file Table1.docx]

Supplementary table 1:

| Variable  (Patient level analysis) | QFR ≤ 0.80 (n=109) | QFR > 0.80 (n=68) | p-value |
| --- | --- | --- | --- |
| On-pump, n (%) | 106 (97.25) | 67 (98.53) | >0.99^1^ |
| Median sternotomy, n (%) | 104 (95.41) | 68 (100) | 0.158^1^ |
| MICS, n (%) | 5 (4.59) | 0 (0%) | 0.158^1^ |
| Robotic assisted surgery, n (%) | 6 (5.50) | 0 (0%) | 0.083^1^ |
| Time on bypass (minutes), median (IR) | 111 (44) | 111 (37.5) | 0.567^2^ |
| Clamp-time (minutes), median (IR) | 69 (36) | 72 (30) | 0.619^2^ |
| ^1^… Fisher`s exact test, ^2^… Mann-Whitney- U test | IR… interquartile range, MICS… minimally invasive coronary surgery, n… number, QFR… quantitative flow ratio | | |
