# Supplementary material for: Limited performance questions retrospective use of quantitative flow ratio in coronary artery bypass grafting
Source: Front Cardiovasc Med. 2026 Feb 2;13:1757011. doi: 10.3389/fcvm.2026.1757011 (PMC12907413; doi:10.3389/fcvm.2026.1757011)
Supplement: Supplementary file 2 [file Table2.docx]

Supplementary table 2:

| **Graft level analysis** | QFR ≤ 0.80 | QFR > 0.80 | p-value |
| --- | --- | --- | --- |
| **LITA** | | | |
| in-situ graft | 58/58 (100%) | 24/24 (100%) | * |
| Y- graft | - | - | * |
| I- graft | - | - | * |
| skeletonized technique | 56/58 (96.55%) | 23/24 (95.83%) | >0.99^1^ |
| **RITA** | | | |
| in-situ graft | 24/26 (92.31%) | 21/22 (95.45%) | >0.99^1^ |
| Y- graft | 2/26 (7.69%) | 1/22 (4.76%) |  |
| I- graft | - | - | * |
| skeletonized technique | 23/26 (88.46%) | 22/22 (100%) | 0.239^1^ |
| **RA** | | | |
| aortocoronary | 36/36 (100%) | 29/30 (96.67%) | 0.455^1^ |
| Y-graft | - | 1/30 (3.33) |  |
| I- graft | - | - | * |
| pedicled technique | 36/36 (100%) | 30/30 (100%) | * |
| ^1^ … Fisher`s exact test  * … p-value not calculable (variabel is a constant)  - … number is zero | | | |
