# Supplementary material for: Limited performance questions retrospective use of quantitative flow ratio in coronary artery bypass grafting
Source: Front Cardiovasc Med. 2026 Feb 2;13:1757011. doi: 10.3389/fcvm.2026.1757011 (PMC12907413; doi:10.3389/fcvm.2026.1757011)
Supplement: Supplementary file 3 [file Table3.docx]

Supplementary table 3:

|  | | RCA | LAD | Diag. | IMED | OM | CX | p-value |
| --- | --- | --- | --- | --- | --- | --- | --- | --- |
| LITA | QFR ≤ 0.80 (n= 58) | - | 58 (100%) | - | - | - | - | * |
|  | QFR > 0.80 (n= 24) | - | 24 (100%) | - | - | - | - |  |
| RITA | QFR ≤ 0.80 (n= 26) | - | 3 (11.54%) | - | - | 16 (72.73%) | 7 (26.92%) | 0.454^1^ |
|  | QFR > 0.80 (n=22) | 1 (4.55%) | 1 (4.55%) | - | 1 (4.55%) | 11 (50.00%) | 8 (36.36%) |  |
| RA | QFR ≤ 0.80 (n=36) | 14 (38.89%) | - | 1 (2.78%) | 1 (2.78%) | 13 (36.11%) | 7 (19.44%) | 0.679^1^ |
|  | QFR > 0.80 (n= 30) | 7 (23.33%) | - | 2 (6.67%) | 3 (10%) | 11 (36.67%) | 7 (23.33%) |  |
| CX… circumflex artery, Diag. … diagonal branch, IMED… Ramus intermedius, LAD… left anterior descending artery, LITA… left internal thoracic artery, n… number, OM… obtuse marginal branch, QFR… quantitative flow ratio, RA… radial artery, RCA… right coronary artery, RITA… right internal thoracic artery  ^1^ Fisher`s exact test  ^*^… p-value not calculable (variabel is a constant)  - … number is zero | | | | | | | | |
