# Supplementary material for: Limited performance questions retrospective use of quantitative flow ratio in coronary artery bypass grafting
Source: Front Cardiovasc Med. 2026 Feb 2;13:1757011. doi: 10.3389/fcvm.2026.1757011 (PMC12907413; doi:10.3389/fcvm.2026.1757011)
Supplement: Supplementary file 4 [file Table4.docx]

Supplementary table 4:

|  | QFR ≤ 0.80 | QFR > 0.80 |
| --- | --- | --- |
| After 1 year | 91.2% ± 2.7% | 83.3% ± 4.4% |
| After 2 years | 91.2% ± 2.7% | 78.9% ± 4.9% |
| After 5 years | 81.3% ± 4.1% | 61.1% ± 6.5% |
