# Supplementary material for: Limited performance questions retrospective use of quantitative flow ratio in coronary artery bypass grafting
Source: Front Cardiovasc Med. 2026 Feb 2;13:1757011. doi: 10.3389/fcvm.2026.1757011 (PMC12907413; doi:10.3389/fcvm.2026.1757011)
Supplement: Supplementary file 5 [file Table5.docx]

Supplementary table 5:

| Variable | HR | 95% CI | p-value |
| --- | --- | --- | --- |
| Age at surgery | 1.020 | 0.988-1.053 | 0.231 |
| Female | 2.444 | 1.276-4.681 | **0.007** |
| Preop. arterial hypertension | 0.820 | 0.431-1.559 | 0.544 |
| Preop. hypercholesterinaemia | 0.670 | 0.345-1.301 | 0.237 |
| Diabetes mellitus:  Absence of DM (reference)  -> Insulin-dependent DM  -> Non-insulin dependent DM | 1.598  0.469 | 0.675-3.783  0.186-1.186 | 0.287  0.110 |
| Smoking status:  Never smoker (reference)  -> former smoker  -> active smoker | 1.799  2.190 | 0.870-3.721  1.173-4.088 | 0.113  **0.014** |
| Preop. adipositas | 0.786 | 0.394-1.568 | 0.494 |
| Preop. PAVD | 0.986 | 0.392-2.480 | 0.975 |
| Preop. COPD | 2.039 | 0.918-4.527 | 0.080 |
| Preop. COPD (inhalative cortisone) | 1.232 | 0.383-3.957 | 0.726 |
| Preop. dialysis | 0.049 | 0.000-54925.5 | 0.671 |
| Preop. TIA | 0.049 | 0.000-1.050*10^16^ | 0.883 |
| Preop. Stroke | 1.067 | 0.259-4.400 | 0.929 |
| Preop. ACS | 0.986 | 0.545-1.784 | 0.964 |
| Preop. PCI | 1.628 | 0.901-2.941 | 0.106 |
| Preop. PCI arterial target vessel | 1.280 | 0.460-3.557 | 0.636 |
| Off-pump | 3.891 | 1.203-12.588 | **0.023** |
| Multiple arterial grafting | 21.158 | 0.010-42808.6 | 0.432 |
| Median sternotomy | 0.527 | 0.127-2.178 | 0.376 |
| Minimally invasive coronary surgery | 1.898 | 0.459-7.848 | 0.376 |
| Time on pump | 0.997 | 0.990-1.005 | 0.455 |
| Clamping time | 1.003 | 0.993-1.013 | 0.610 |
| Skeletonized harvesting technique | 2.411 | 1.285-4.523 | **0.006** |
| Percentage target vessel stenosis (%) | 0.988 | 0.975-1.002 | 0.084 |
| Percentage target vessel stenosis ≥ 70% | 0.369 | 0.146-0.932 | **0.035** |
| Percentage target vessel stenosis ≥ 80% | 1.534 | 0.800-2.944 | 0.198 |
| Percentage target vessel stenosis ≥ 90% | 1.133 | 0.642-1.998 | 0.667 |
| Percentage target vessel stenosis ≥ 99% | 0.750 | 0.401-1.401 | 0.366 |
| Left main stenosis ≥ 50% | 0.877 | 0.396-1.943 | 0.747 |
| QFR ≤ 0.80 | 0.528 | 0.310-0.899 | **0.019** |
| Postop. antiplatelet therapy | 0.109 | 0.015-0.815 | **0.031** |
| Postop. DAPT | 1.266 | 0.596-2.688 | 0.539 |
| Postop. Statin therapy | 1.280 | 0.509-3.218 | 0.599 |
| Postop. Ezetimibe therapy | 2.401 | 1.119-5.153 | **0.025** |
| Postop. PCSK9I therapy | 37.385 | 4.367-320.019 | **<0.001** |
| Postop. beta-blocker | 0.771 | 0.424-1.402 | 0.394 |
| Postop. insulin | 1.787 | 0.759-4.207 | 0.184 |
| Postop. oral antidiabetic therapy | 0.437 | 0.174-1.098 | 0.078 |
| ACS…acute coronary syndrome, CI…confidence interval, COPD… chronic obstructive pulmonary disease, DAPT… dual antiplatelet therapy, DM…diabetes mellitus, HR… hazard ratio, PAVD… peripheral arterial vascular disease, PCI… percutaneous coronary intervention, PCSK9I… proprotein convertase subtilisin/kexin type 9 inhibitor, postop. … postoperative, preop. … preoperative, QFR… quantitative flow ratio, TIA… transitory ischaemic attack | | | |
