# Supplementary figures and images for: Limited performance questions retrospective use of quantitative flow ratio in coronary artery bypass grafting
Source: Front Cardiovasc Med. 2026 Feb 2;13:1757011. doi: 10.3389/fcvm.2026.1757011 (PMC12907413; doi:10.3389/fcvm.2026.1757011)

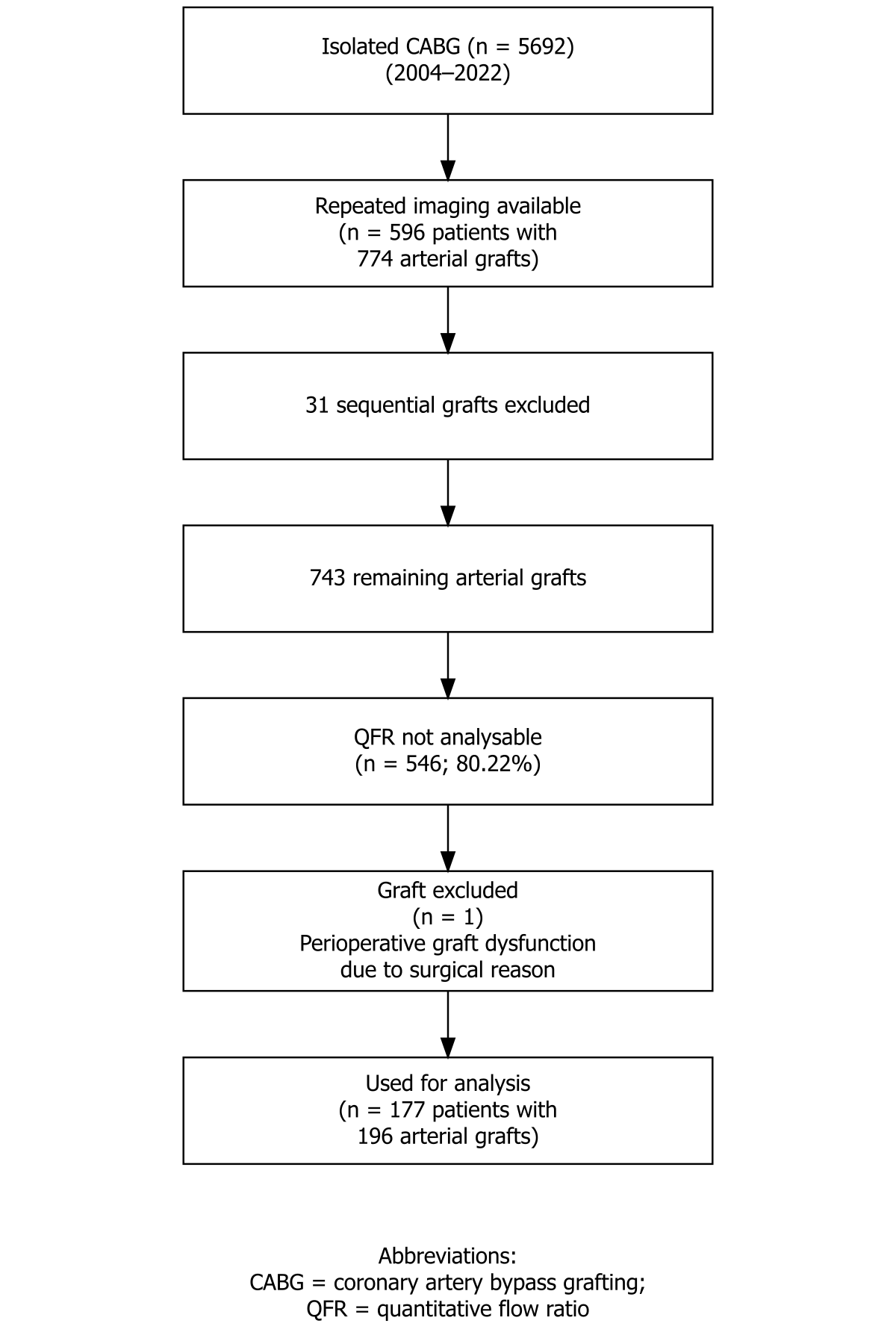

Supplement: Supplementary file 6 [file Image1.tif]
